# Environmental Effects of China’s Export Trade to the Countries along Belt and Road: An Empirical Evidence Based on Inter-Provincial Panel Data

**DOI:** 10.3390/ijerph20064698

**Published:** 2023-03-07

**Authors:** Juan Huang, Kai Zhang, Hui Zhao, Rong Fu, Zhiguo Li

**Affiliations:** 1 School of Economics, Hang Zhou Dian Zi University, Hangzhou 310018, China; 2 School of Economics, University of Essex, Essex CO4 3SQ, UK; 3 School of Economics and Management, Wuhan University, Wuhan 430000, China

**Keywords:** belt and road, export trade, environmental impact, CO_2_ emission

## Abstract

There is a close inherent connection between manufacturing exports and environmental pollution. With the continuous growth of China’s export trade to the countries along Belt and Road, the resulting environmental problems have also received much attention. This paper first analyzes the environmental impact mechanism of China’s export trade to the countries along Belt and Road. Then based on the dynamic panel data of 30 provinces in China from 2013 to 2019, we use the SYS-GMM method to make an empirical test from national and regional perspectives and analyze the environmental effects of China’s export trade to the countries along Belt and Road. The results show that the environmental effects of export trade are significantly heterogeneous in different regions. In general, export trade has a significant positive scale effect on CO_2_ emissions; the negative effect of environmental regulation on CO_2_ emissions can effectively offset the positive effect caused by the growth of output in the capital-intensive sector, and the composition effect is generally negative; the technical effect of China’s export trade to the countries along Belt and Road mainly depends on the technological-independent innovation, which is caused by the domestic investment in science and technology, so the overall technical effects are negative. Therefore, China should optimize the structure of export trade, promote technological innovation, and cultivate green advantage industries by increasing investment in scientific research and development; implement a gradient environmental regulation policy; and improve the quality and level of foreign direct investment.

## 1. Introduction

As global value chains continue to deepen adjustment, the Belt and Road Initiative has provided new impetus to the growth of trade for China, the countries along Belt and Road and even the whole world since it was proposed in 2013. The scale of China’s export trade to the countries along Belt and Road has been increasing year by year, and according to the data from the Statistical Communique of China’s National Economic and Social Development, the total value of China’s exports was CYN 21.7348 trillion in 2021, and China’s exports to the countries along Belt and Road amounted to CYN 6.5924 trillion, accounting for 30% of the country’s total exports. The export commodities are mainly manufactured goods, with the characteristics of manufacturing export oriented. Manufacturing industries are the main prat in producing all kinds of pollution. In the world of environmental history, large-scale pollution problems are concentrated in the period of rapid development of industrial production [1]. Great attention has been paid to whether the growing trade activities in the Belt and Road regional value chain will aggravate pollution as the world’s largest carbon emitter and manufacturing trading country. According to the report published by the International Energy Agency (IEA) in 2021, China is the world’s largest emitter of carbon with a carbon dioxide emission of 11.9 billion tons in 2021 accounting for 33% of the global total emissions. China has been facing increasingly serious environmental pollution and resource waste due to the continuous expansion of its extensive and energy-intensive traditional export industries in the early and middle stages of industrialization and opening up. Since 2010, China has entered the middle and late stages of industrialization development [2]. The development of the manufacturing industry and export trading is badly needed to upgrade. The carbon intensity of most regions along the Belt and Road is significantly higher than the global average level [3], which makes it necessary to build a green Belt and Road. Therefore, there is an important, realistic issue that needs to be solved: How can China improve the quality of export trade and reduce carbon emissions by promoting the construction of the green Belt and Road Initiative? Furthermore, what will be the environmental effects of China’s exports to countries along the Belt and Road? Through which specific pathways does export trade impact carbon dioxide emissions? How to realize the green transformation and export trade upgrading? The research on the above issues is not only conducive to promoting the construction of the green Belt and Road Initiative but also has important theoretical and practical significance for China to realize upgrading export trade in a high-quality and low-carbon way.

## 2. Literature Review

There is a deep relationship between international trade research and environmental pollution. Many studies generally believe that trade liberalization will raise pollution in the short term, but there is some debate about whether the effects raise or lower pollution in the long term. The Environmental Kuznets Curve (EKC) [4], the Pollution Haven Hypothesis (PHH) [5] and other theories have been gradually developed in the process of disputes, and these hypotheses themselves have become a new focus of contention. The Environmental Kuznets Curve varied with different empirical research, including a lot of support and opposition of results. The conclusion showed that the economic development stage of a country and sample data in short or long term would influence the outcome of empirical testing. Some scholars supported the establishment of EKC by empirical research [6,7,8]. Zafeiriou et al. conducted an empirical test on whether the Environmental Kuznets Curve (EKC) exists in the three newly joined European Union countries: Bulgaria, Czech Republic and Hungary. They examined the relationship between carbon emissions per thousand hectares of agriculture and the economic performance of the agricultural sector. The results showed that the EKC hypothesis in Bulgaria and Czech Republic was confirmed in the long term, while it only existed in the short term in Czech Republic [8]. There are also scholars who argue that the Environmental Kuznets Curve (EKC) does not exist. Dogan and Turkekul studied the relationship between carbon dioxide emissions, energy consumption, real output (GDP), square of real output, trade openness, urbanization and financial development in the United States from 1960 to 2010. The results showed that the EKC hypothesis does not hold in the United States, as real output promotes environmental improvement while increasing gas emissions [9]. Gill et al. tested the existence of the EKC curve in greenhouse gas emissions in Malaysia using carbon dioxide emission data from 1970 to 2011. They found that greenhouse gas emissions increase with economic growth, and the insignificant coefficient of GDP square does not support the turning point of the EKC curve indicating that only continued economic growth can reverse the trend of environmental degradation in Malaysia [10]. Li et al. empirically analyzed the relationship between air quality and economic growth in the Beijing-Tianjin-Hebei region from 2006 to 2017. The results showed that the Environmental Kuznets Curve for air quality in the Beijing-Tianjin-Hebei region is inverted “N” shaped but has not reached the turning point. Industrial structure has a negative impact on air quality, while population density, urbanization and urban greening have a positive effect on improving air quality, but increasing urban greening has no significant effect on improving air quality [11]. 

The Pollution Haven Hypothesis, first put forward by Walter and Ugelow [5], is an important part of the Environmental Kuznets Curve theory. The hypothesis is that as restrictions on trade and foreign investment are reduced, polluting economic activities may shift from countries with strict environmental regulations to those with light or no regulations, and some countries may use lax environmental regulations as a competitive strategy to attract foreign investment. Copeland and Taylor preliminarily validated the existence of pollution havens by constructing a static North–South general equilibrium model of trade. [12]. They proposed that international trade would promote the transfer of pollution-intensive industries from developed countries to developing countries, which have more loose environmental regulations and become pollution havens under the condition of an open economy. Copeland and Taylor distinguished between the pollution haven effect and the pollution haven hypothesis. The pollution haven effect referred to the phenomenon where countries with strict environmental regulations that import pollution-intensive products will adopt even more stringent environmental standards, leading to increased specialization in producing clean products [13]. Cole argued that the pollution haven hypothesis could explain the traditional EKC curve in developed countries as these countries specialized more in the service and light manufacturing sectors while gradually reducing heavy industry production. However, this may not happen in developing countries where there may not be other places to absorb industrial transfers, especially when their competitiveness relies on specialization in heavy industry production. Therefore, developing countries were prone to becoming “pollution havens” under free trade, where the local economy developed at the expense of environmental degradation [14].

The Pollution Haven Hypothesis has been supported by many scholars [15,16,17,18]. Yang and Tian found that pollution havens existed in the eastern, central and western regions of China, and foreign investment increased the risk of pollution-intensive industries moving to China. As a result, the eastern region of China was most severely affected by environmental pollution [17]. Zhang et al. found that pollution transfer existed in China’s export trade to the countries along Belt and Road, that is to say some highly polluting industries were transferred to the countries along Belt and Road, thus improving China’s local environmental conditions to a certain extent, but the environmental problems faced by the countries along Belt and Road could not be effectively solved [18]. 

However, some scholars have conducted empirical research using different pollution indicators, nationalities and methods, and the results show that the pollution haven hypothesis is not valid [19,20,21,22]. Eskeland and Harrison investigated the relationship between trade and environmental standards in the United States and Canada and found that differences in environmental regulations can lead to pollution transfer. At the same time, the paper also found evidence that coordinating and unifying environmental standards could effectively curb pollution transfer [20]. Beine et al. examined the pollution haven hypothesis using a quasi-natural experiment based on the adoption of the Euro 5 emission standards in Europe. They found that stricter environmental regulations did not lead to pollution havens, and that there was no evidence that firms would migrate to countries with lower environmental standards [22].

According to the theory of trade complexity, the environmental effects are comprehensive, which depends on many aspects. Many scholars put forward analysis frameworks of environmental effects in different perspectives. Grossman and Krueger [4] first proposed the inverted U-shaped Environmental Kuznets Curve (EKC) and established the basic theoretical framework of international trade environment effects, including scale effect, composition effect and technique effect. Later, several scholars found that beyond the three effects above, the environmental effects also included product effect [23], policy effect [24] and income effect [25]. In general, the three effects of the environment theoretical framework is the most representative and widely accepted. After that, many scholars conducted empirical studies based on this theoretical framework. Antweiler et al. combined factor endowment theory to construct an empirical model that comprehensively considers the scale effect, composition effect and technique effect of trade on the environment. The scale effect was measured by indicators, such as trade openness, per capita income and population density. The composition effect was measured by indicators, such as the export ratio, substitution elasticity of imported goods and export elasticity of goods. The technique effect was measured by indicators, such as the capital–labor ratio. The model was tested with sulfur dioxide concentration data, and the results supported the theoretical model. It showed that the changes in a country’s output composition due to trade had a relatively small impact on pollution, while the technique effect and scale effect caused a net decrease in pollution sources. Therefore, overall free trade was beneficial for reducing environmental pollution intensity [26].

Afterwards, the ACT model was improved by many scholars [27,28,29,30,31,32,33]. Hu and McKitrick used the ACT model to study how international trade affects pollutant emissions when pollutants were by-products of consumption and pollution-intensive products and found that the scale effect and technique effect were positive in general [34]. Nasir et al. studied the data of five ASEAN countries from 1982 to 2014 and found that economic growth, FDI and financial development would promote domestic carbon emissions [35]. Salman et al. compared the impact of the import and export trade of seven ASEAN countries on carbon emissions and found that the trade of seven ASEAN countries had technique effects and reduced carbon emissions [36]. Wang et al. analyzed the data from 2005 to 2014 of 64 countries (or regions) and found that in the early stage of green Belt and Road construction, each economy was likely to move up the value chain and achieve carbon emission reduction [37].

Through the above analysis, it can be seen that EKC, PHH and the three effects theory of the environment explain the specific effects from the perspectives of time dimension, space dimension and decomposition quantification, respectively. The literature provide some important references for this paper. There are few studies on the environmental effects of economic and trade development in the South–South region. However, the South–South trade within the Belt and Road region has become a new growth point of global trade, and the pollution emission problem has become the focus of attention. Different levels of economic development, geographical location, industrial structure and policies in the Belt and Road region may produce different environmental effects. This paper explores the environmental effects of China’s export trade to the countries along Belt and Road from overall and regional perspectives, tests the relationship between export trade and pollution emission and proves whether the environment of Kuzshanes curve exists. In the construction of the econometric model, we also take into account the level of environmental regulation so as to verify whether different environmental standards will lead to the relocation of polluting industries, and thus produce the pollution paradise effect. It will enrich the empirical study on the environmental effects of foreign trade and provide references for the formulation and implementation of relevant regional trade and environmental policies.

## 3. Theoretical Mechanism

According to Grossman and Krueger’s theory, total pollution emissions depend on scale effect, structure effect and technology effect. This paper analyzes the CO_2_ emission effect mechanism of export trade under the green Belt and Road Initiative by adding the impact of environmental policies and input of imported intermediate goods. The specific mechanism is as follows:

Firstly, scale effect reflects the CO_2_ emissions brought by the expansion of production scale caused by export trade. The conclusion that the expansion of production scale or economic scale of trade leads to the increase of CO_2_ emissions has been confirmed by a large number of studies [38,39]. With the inclusive development of the Belt and Road Initiative, there is an increasing growth in intra-regional trade and the continuous increase of China’s export trade to the countries along the Belt and Road has directly led to the expansion of its domestic industry. Although it increases resource consumption and carbon emissions, it has also raised social environmental awareness, which has led to high environmental quality needs and prompted the government to formulate stricter environmental policies [25]. The increase of economic income and average income lever also support carbon emission reduction. Therefore, scale effect is the result of a combination of the above factors.

**Hypothesis** **1.** *China’s export trade to countries along the Belt and Road will increase CO_2_ emissions through scale effect.*

Secondly, composition effect reflects the influence of industrial structure change induced by export trade on CO_2_ emissions. Under the Belt and Road Initiative, a country’s comparative advantage is the determinants when it participates in value chain division and trade. If the advantageous industries or production stage that China’s export trade to countries along the Belt and Road was pollution-intensive, China’s export trade would occupy a higher proportion in industrial structure and the CO_2_ emissions would increases accordingly while the composition effect is negative. On the contrary, if the export increase of advantageous products or product production stage is clean, it will reduce carbon emissions and bring a positive composition effect. The industrial structure will be upgraded to low-carbon direction.

The essence of the Belt and Road export trade is complementary industrial advantages between China and countries along the Belt and Road. In the long run, the development of clean industries or product production stages with high technology, high added value and low pollution under the green Belt and Road Initiative will become the goal of policy orientation and enterprise transformation and upgrading. Industrial transfer is conducive to promoting the upgrading of China’s industrial structure towards high technology, high added value and low carbon and promoting the long-term reduction of carbon emissions [40]. On the other hand, the environmental standards for imported products in southern markets are generally lower than those in northern markets. Due to the low per capita income level, consumers do not pay enough attention to environmental protection and have low requirements on product quality and diversification. Therefore, in order to gain more profits, manufacturers will adopt relatively simple and low-end production processes and technologies, which is not conducive to reducing carbon emissions. In addition, the government’s environment regulation policies steer the structural adjustment of export trade. All environmental impacts of trade are closely related to government policies, thus positive policies ensure that trade liberalization can ultimately improve welfare [24]. It can be seen that the results of composition effect in different regions are not the same.

**Hypothesis** **2.** *The carbon emission composition effect of China’s export trade to countries along the Belt and Road will show regional heterogeneity.*

Finally, technique effect reflects the impact of the progress of production technology on CO_2_ emissions. Under the green co-construction concept of the Belt and Road Initiative, various green trade and environmental policies help promote the innovation, upgrading and diffusion of green technologies, which will have a positive impact on CO_2_ emissions. On the one hand, with the advantages of resources and labor factors gradually disappearing, in order to obtain sustainable profits and higher added value, enterprises need to foster new export competitive advantages to participate in the division of the Belt and Road value chain and trade. Policies and R&D investment characterized by green industrial development and green technological innovation are conducive to promoting technological innovation and green upgrading of enterprises. What’s more, enterprises possess internal innovation resources by early capital and experience accumulation. Additionally, the generation and development of new technologies is conducive to improving production efficiency and improving environment pollution. On the other hand, under the requirement of high-quality development, the government should not only focus on quantity growth but also on quality when introducing foreign investment. By focusing on introducing foreign capital with advanced and clean technology and guiding it in advantageous industries, we can promote technology transfer and produce technology spillover effects. Eventually production efficiency and the cultivation rate of green advantage industries can improve, as well as CO_2_ emissions being be reduced. Furthermore, in the era of global value chains, China’s participation in the value chain division of labor is deepening. The growth of export trade will promote the increase in demand for imported intermediate goods in related industries, and the imported intermediate goods may be high-tech service goods or technology-intensive, which is conducive to upgrading the quality of export products [41] and brings about technology spillover effects that cannot be ignored. Therefore, the technique effect is most likely to have a positive effect in the impact mechanism of export trade on CO_2_ emissions.

**Hypothesis** **3.** *China’s export trade to countries along the Belt and Road will reduce CO_2_ emissions by promoting technological progress.*

In summary, China’s export trade to the countries along the Belt and Road will impact on the environment through the expansion of domestic production scale, promotion of social environmental awareness, stricter environmental regulations, adjustment of industrial structure and green technology innovation, upgrade and diffusion, etc. Therefore, the environmental effect is a result of multiple factors and the specific effect cannot be inferred from economic and social phenomena alone, and there is an increasing need for empirical evidence.

## 4. Construction of Econometric Models and Data Sources and Variable Specification

### 4.1. Construction of Econometric Models

Based on the above theoretical mechanism analysis, this paper refers to the theoretical model of three effects proposed by Grossman and Krueger [4], and the formula is shown as follows:

*Z* = Σ *s_i_ e_i_ X* (1)

where *Z* represents the pollution emissions caused by export trade, *s_i_* represents the proportion of exports of sector *i* in total exports, *e_i_* represents the pollution intensity of sector *i* and *X* represents the total exports.

The empirical model of environmental effects constructed by Antweiler et al. [26] is constructed as follows:

Ln*E_it_* = *α* + *β*_1_Ln*GDP_it_* + *β*_2_Ln*POP_it_* + *β*_3_Ln*GDP_pcit_* + *β*_4_Ln*KL_it_*/*L_it_* + *β*_5_Ln*IMP_it_* + *β*_6_Ln*EXP_it_* + *β*_7_*DUMMY_it_* + *ε_it_* (2)

where Ln*E_it_* represents the environmental effect of country *i* at time *t*; Ln*GDP_it_*, Ln*POP_it_* and Ln*GDP_pcit_*, respectively, represent the natural logarithm of the real *GDP*, total population and per capita *GDP* of country *i* at time *t*; Ln*KL_it_*/*L_it_* represents the natural logarithm of the capital–labor ratio of country *i* at time *t*. Ln*IMP_it_* and Ln*EXP_it_* represent the natural logarithms of imports and exports of country *i* at time *t*; *DUMMY_it_* is a dummy variable indicating whether the country *i* is a high-income country at time *t*; *α* and *β*_1_–*β*_7_ are the coefficients of the model; and *ε_it_* is the error term.

Based on (1) and (2), considering the continuous and dynamic adjustment of pollutant emissions, this paper relaxes the assumptions of the static model and adds the first-order lag term for per capita CO_2_ emissions. The intensity of environmental regulation and imported intermediate inputs are also taken into account to construct a dynamic model for the environmental effects of China’s export trade to the countries along Belt and Road. The specific benchmark regression model is set as follows:

Ln*C_it_* = *β*_0_ + *β*_1_Ln*C_it_*_−1_ + *β*_2_Ln*T_it_* + *β*_3_Ln*I_it_* + *β*_4_(Ln*I_it_*)^2^ + *β*_5_*K_it_*/*L_it_* + *β*_6_Ln*R_it_* + *β*_7_Ln*STEI_it_* + *β*_8_Ln*M_it_* + *β*_9_Ln*FDI_it_* + *λ_it_* + *η_it_* + *ε_it_* (3)

where *i* and *t* represent the province and year, respectively; *C_it_* represents per capita CO_2_ emissions; *C_it_*_−1_ represents the first-order lag term of per capita CO_2_ emissions, considering the correlation of CO_2_ emissions, the first-order lag term of the dependent variable is added to the right of the reference equation; *T_it_* represents the export trade openness to countries along the Belt and Road; *I_it_* represents per capita income levels; (*I_it_*)^2^ represents the quadratic term of per capita income levels; *K_it_*/*L_it_* represents the capital–labor ratio; *R_it_* represents environmental regulation intensity; *STEI_it_* represents science and technology expenditure intensity; *M_it_* represents input of imported intermediate products; *FDI_it_* represents foreign investment level; *λ_i_* represents fixed effect of provinces; *η_t_* represents time fixed effect; *ε_it_* represents random disturbance term; and *β*_0_, *β*_1_, …, *β*_9_ represent the estimated parameters. In order to reduce the possible effects of heteroscedasticity and time trend factors, all variables were logarithmically processed to make the data more stable and easier to analyze.

### 4.2. Data Sources

Considering the availability of data and better observation the influence of the Belt and Road Initiative unveiled in 2013, this paper selected the inter-provincial dynamic panel data of 30 provinces in eastern, middle and western China from 2013 to 2019 as research samples. Regional classification standards refer to the Big Data Report on Belt and Road Trade Cooperation. In order to facilitate data processing, we combine Northeast China into Central China for analysis. Then, the eastern region includes Beijing, Tianjin, Hebei, Shanghai, Jiangsu, Zhejiang, Fujian, Shandong, Guangdong and Hainan; the middle region includes Shanxi, Liaoning, Jilin, Heilongjiang, Anhui, Jiangxi, Henan, Hunan and Hubei; the western region includes Inner Mongolia, Guangxi, Chongqing, Yunnan, Guizhou, Sichuan, Shaanxi, Gansu, Qinghai, Ningxia and Xinjiang. All of the original data in this paper are obtained from statistical yearbooks and environmental yearbooks by provinces, the Big Data Report on Belt and Road Trade Cooperation, China Statistical Yearbook, China Industrial Economy Statistical Yearbook, China Environmental Statistical Yearbook, China Belt and Road Official website, China Research Website, etc. The CO_2_ emissions data for each province is calculated by WRI based on the provincial energy balance sheet data in the China Energy Statistical Yearbook.

### 4.3. Variable Specification

(1) CO_2_ emissions (Ln*C_it_*). In this paper, per capita carbon dioxide emission is selected as the explained variable of pollutant, and the first-order lag term is introduced as the dynamic term. Since China’s export commodities to the Belt and Road are mainly manufactured goods and the energy consumption of the manufacturing industry accounts for more than half of the whole society’s energy consumption, which is the main source of CO_2_ emissions, it is of representative significance to use CO_2_ as the sample data of pollutant.
(2) Export trade openness (Ln*T_it_*). The export trade openness of each province is represented by the proportion of its export trade to the countries along the Belt and Road in its GDP, with the purpose of examining whether the export trade of each province to the Belt and Road affects the CO_2_ emissions in terms of scale.
(3) Per capita income levels (Ln*I_it_*). According to the EKC hypothesis, income level is an important control variable that affects CO_2_ emissions. Thus, this paper uses the average wage of employees in each province to measure the level of per capita income. A large number of studies have found an inverted U-shaped relationship between pollution emissions and per capita income [6,27], so the introduction of the logarithmic value of per capita income (Ln*I_it_*) and the logarithm of the quadratic term of per capita income ((Ln*I_it_*)^2^) is to examine whether the scale and technique effects of economic growth meet the EKC hypothesis.
(4) Capital–labor ratio (Ln*K_it_*/*L_it_*). For carbon dioxide air pollutants, higher Kit/Lit means higher output in capital-intensive sectors, and thus more carbon emissions from production. Therefore, *K_it_*/*L_it_* can be used to reflect the environmental effect caused by the production structure changes, which representing structural effects. Referring to the practice of Sheng and Lv [21], the capital of each province is expressed by the average annual balance of the net value of industrial fixed assets of the whole society, and the labor force is expressed by the average number of employed persons.
(5) Environmental regulation intensity (Ln*R_it_*). It is generally believed that strict environmental regulations are conducive to improving the quality of the environment, while looser environmental regulations not only lead to increase CO_2_ emissions from domestic companies but also make them become “pollution paradise” of other countries, resulting in more serious environmental pollution. We use the logarithm value Ln*R_it_* of carbon tax set by government representing the composition effect to measure the level of environmental regulation, aiming to examine whether the difference in sewage charges would have a significant impact on changes of industrial structure.
(6) Science and technology expenditure intensity (Ln*STEI_it_*). Referring to the practice of Hu Yi et al. [32], we select the proportion of science and technology expenditure of local governments’ financial expenditure to express the intensity of science and technology expenditure and measure the level of technology research development of this province. Science and technology level, representing the technique innovation effect of environmental impact, is the basis of the comprehensive economic strength of each province, which affects local products’ international competitiveness and reduces CO_2_ emissions.
(7) Input of imported intermediate products (Ln*M_it_*). China, as an important part in the Belt and Road value chain and the middle of global intermediate trade, imports intermediate products, such as parts and components, which probably brings technique spillover effects. In the process of importing intermediate goods containing more advanced technologies, developing economies tend to relearn and imitate in order to reduce import costs so as to obtain technological progress [42]. Therefore, this paper selects the logarithm value Ln*M_it_* of import intermediate products in each province to represent the technique spillover effect on the environment.
(8) Foreign investment level (Ln*FDI_it_*). Under the productive globalization, FDI is one of the decisive factors for exports. On the one hand, FDI can promote the technological progress of the host country through the introduction of clean technologies and technology diffusion effects, thereby improving production efficiency, optimizing resource allocation and reducing CO_2_ emissions. On the other hand, it is also possible to increase CO_2_ emissions from industrial production in the host country through the transfer of polluting industries. We choose the numerical value of the foreign direct investment flow of the countries along the Belt and Road to Chinese provinces to represent the technological effect of the new foreign direct investment on carbon emissions since the proposal of the Belt and Road Initiative.

## 5. Empirical Analysis and Results

In a dynamic panel data model, the use of lagged dependent variables as explanatory variables may lead to correlation between explanatory variables and random disturbances, as well as cross-sectional dependence. As a result, traditional estimation methods are likely to generate biased and inconsistent parameter estimates, distorting the economic implications inferred from these parameters. To address these issues, Blundell and Bond proposed the system GMM method, which effectively solves the aforementioned problems [43]. Therefore, we use the systematic GMM method to estimate Model (3). Compared with differential GMM estimation, system GMM estimation combines the difference equation and the level equation as an equation system, which improves the accuracy of the analysis results. Therefore, we use a two-step method to estimate the dynamic panel model from the whole country, the eastern, the middle and the western regions, respectively, and the Hausman test is used to examine the effectiveness of the over-identifying restrictions, which can determine the robustness of the GMM estimates. Then the Arellano–Bond statistic is used to test for serial correlation of difference error terms. The consistency of the GMM estimator requires that there is no second-order sequence correlation in the difference equation, but the first-order sequence correlation is allowed. The specific regression results are shown in Table 1.

From the estimation results of the whole country and each region, the Hausman test does not reject the null hypothesis; it indicates that the selected instrumental variables are reasonable, and we should adopt the fixed effect model. The *p* values of AR (2) are not significant, which indicates that the four model estimators accept the null hypothesis that there is no sequence correlation, and the moment constraint of the difference equation is reasonable. The lagging period CO_2_ emissions index Ln*C_it_*_−1_ is significantly positive at the 1% level showing that the dynamic model setting in this paper is effective, and the adjustment of CO_2_ emissions is indeed a continuous and cumulative process.

(1) The effect of export trade openness (Ln*T_it_*). The Ln*T_it_* coefficient is significantly positive at a level of 1% from the estimation results of the national and regional models, which shows, to some extent, that the increasing export trade openness has increased CO_2_ emissions and manifests the growth of China’s export trade to the Belt and Road countries generates more CO_2_ emissions. From the perspective of various regions, the export trade openness index is the most significant impact on the middle region, followed by the western region, and the eastern region has the least impact. Although from the perspective of the regional distribution of export trade China to the Belt and Road, the eastern region occupies the largest proportion and is rising fastest. However, the eastern region’s exports to the Belt and Road occupies the lowest proportion in this region’s export trade. The west region has the highest proportion, followed by the middle region. This phenomenon should be directly related to the impact of the export trade openness on CO_2_ emissions.
(2) The effect of per capita income levels (Ln*K_it_*/*L_it_*). From the perspective of the country and the eastern region, the primary term coefficient of per capita income is significantly positive, and the quadratic term coefficient is significantly negative, which indicates that there is an inverted U-shaped EKC relationship between national and eastern regions’ per capita income and pollution emissions. The scale and technique effect of economic growth meet the EKC hypothesis, which shows that from the perspective of the country and the eastern region, the per capita income level has exceeded the inflection point of the inverted U-line, that is, with the increase of people’s income level, the environmental quality will have higher requirements to promote reductions in pollution emissions. However, there is no inverted U-shaped relationship in the middle and western regions. The first-order coefficient of per capita income is significantly positive at the level of 5%, but the effect of the second-order coefficient is not significant, indicating that the increasing economic scale and level of per capita income have increase CO_2_ emissions.
(3) The effect of the capital–labor ratio (Ln*K_it_*/*L_it_*). It can be seen from Table 1 that the capital–labor ratio indicators of the country and the east, middle, and west have significantly positive effects. An increase in the capital–labor ratio means an increase in output in capital-intensive sectors, thereby increasing CO_2_ emissions from production. According to the data, China’s exports to the countries along the “Belt and Road” are mainly manufactured products, of which 57.9% are exports of mechanical and electrical products, and 28.3% are exports of high-tech products, indicating that the dominant products China’s export trade to the Belt and Road are still mainly pollution-intensive and resource-intensive industrial industries. Changes in the industrial structure caused by the growth of export trade have increased energy consumption and environmental pollution. The eastern region has the most significant effect, and the middle and western regions are relatively weak. This should be related to the industrial structure and the characteristics of advantageous industries. The eastern region is mainly dominated by the secondary and tertiary industries when the middle and western regions are still dominated by the primary industry and their scale of export trade is relatively small, so its influence on CO_2_ emissions is weak.
(4) The effect of environmental regulation intensity (Ln*R_it_*). From the perspective of the whole country and the eastern region, the environmental regulation policy has a significant negative effect on CO_2_ emissions at the level of 1%, especially in the eastern region, given that the environmental regulation policy is always the most stringent when the region has strong economic strength. Therefore, the emission reduction effect of environmental regulation intensity is the most significant; the environmental regulatory policies in the middle and western regions show a negative effect at the level of 10%. In general, the economic levels of the middle and western regions are relatively low and the demand for economic growth is higher than the demand for environmental quality. Therefore, the intensity of environmental regulations is relatively low and enforcement efforts are weak. In addition, the level of industrialization is not high, so the effect of environmental regulations on CO_2_ emission reduction is not as obvious as in the eastern region.
(5) The effect of science and technology expenditure intensity (Ln*STEI_it_*). From the national and eastern, middle, and western estimates, the coefficients on the intensity of scientific and technological expenditures are both significantly negative at the level of 1%, and the effects in the national and eastern regions are particularly significant. It shows that the increase in the intensity of scientific and technological expenditure will promote the improvement of scientific and technological innovation levels, thereby effectively improving the development and use of cleaner production technologies and reducing industrial pollution emissions in the production process. Due to regional differences in the level of economic development, the eastern region has the highest level of industrialization and stricter environmental regulations so it faces the greatest pressure of technology upgrading. The annual investment in scientific and technological innovation is higher than that in the central and western regions, which brings about significant effect of technological progress and technological innovation and the best effect of carbon emission reduction.
(6) Input of imported intermediate products (Ln*M_it_*). It can be seen from Table 1 that the input coefficients of imported intermediate products in the country and the middle and western regions are not significant, and imported intermediate products have no significant technological spillover effect on environmental pollution emissions. The main reason may be that China’s export trade to the “Belt and Road” countries is dominated by general trade, and processing trade is relatively small, so there is less demand for intermediate input; on the other hand, it indicates that most of China has not yet achieved globalization. The high-value-added links of the value chain are climbing, and processing trade is still mainly labor intensive, especially in the middle and western regions. The eastern region has a significant negative effect at the 5% level. For every 1% increase in the input of imported intermediate products, CO_2_ emissions will decrease by 0.189%, indicating that there is a certain technique spillover effect of imported intermediate products in the eastern region. The relatively high technological content of export products in the region is related.
(7) Foreign investment level (Ln*FDI_it_*). Judging from the indicators of foreign investment levels in the country and in the eastern, middle, and western regions, neither of them has a significant effect. It is clear that the Belt and Road countries have not brought significant technique spillover effects to China’s FDI, and foreign investment has no obvious impact on CO_2_ emissions. From the FDI data of countries along the Belt and Road to China, we can see that only the developed countries with high FDI levels in China are South Korea and Singapore, and the countries along the Belt and Road have frequent fluctuations in FDI and have great instability. This shows that the quality of FDI investment in China’s introduction of the Belt and Road countries needs to be further improved.

## 6. Conclusions, Policy Recommendations and Future Research Directions

### 6.1. Conclusions

Based on the dynamic panel data of China’s 30 provinces from 2013 to 2019, we empirically test the environmental effects of China’s export trade on the countries along the Belt and Road from various regions in the whole country, eastern, middle and wester and draw the following main conclusions:

1. From the perspective of scale effect, an increase in the openness of export trade will increase CO_2_ emissions, of which the middle region has the most significant impact, followed by the western region, and the eastern region has the least impact. The increase in the scale of trade directly increases the amount of CO_2_ emissions, resulting in a direct positive scale effect; another indicator to measure the scale effect—the level of per capita income and the impact of pollution emissions show different results in different regions. In the national and eastern regions, there is an inverted U-shaped EKC relationship between per capita income and pollution emissions, which shows that the final result of export trade is to drive economic growth and increase income levels to guide society as a whole to an environmentally friendly development, thereby bringing negative impacts. There is no inverted U-shaped relationship in the middle and western regions for the scale effect. The increase in per capita income has not raised people’s requirements for environmental quality. Instead, export trade has promoted the expansion of the economic scale and caused more pollution. Emissions show a positive scale effect. In general, the scale effect in the middle and western regions is significantly positive, while the negative effect brought by the per capita income level in the national and eastern regions is not enough to offset the positive effect caused by the growth of export trade, so the scale effect is also positive.
2. From the perspective of composition effects, on the one hand, due to the uneven regional development of China, the difference between each region’s industrial structure is obvious. From the perspective of China and the eastern, middle, and western regions, the capital–labor ratio has a significant positive effect on CO_2_ emissions. The eastern region has the most significant effect, and the middle and western regions are relatively weak. Mainly because the eastern region takes the lead in development and completes capital accumulation first. So it is more likely to obtain comparative advantages in capital-intensive sectors. The fact verifies that the dominant industries of the Belt and Road export trade are pollution-intensive and resource-intensive industries, and changes in the industrial structure caused by the growth of export trade have increased environmental pollution, thereby showing a positive structural effect. On the other hand, environmental regulation policies have different effects on different places. From the perspective of the country and the eastern region, the intensity of environmental regulations has a significant negative effect on CO_2_ emissions. Strict environmental regulation policies have the most significant effect on reducing emissions in the eastern region, while environmental regulation policies in the middle and western regions are conducive to reducing where the effect of CO_2_ emissions is not as obvious as in the eastern region. In general, the government’s environmental regulatory policies are conducive to reducing CO_2_ emissions and have a negative composition effect. In summary, the negative effect caused by strict environmental regulation policies can effectively offset the positive effect caused by the output growth of capital-intensive sectors, so the composition effect is negative.
3. As for the technique effect, from the estimation results of the country and the east, middle and west, the increase of scientific and technological expenditure intensity is conducive to significantly reducing CO_2_ emissions, and the impact is most prominent in the eastern region, indicating that scientific and technological research and development investment can be effectively transformed into science and technology. The innovation ability promotes the development and application of green production technology, thereby reducing CO_2_ emissions and showing a negative technique effect, while the technique spillover effect of imported intermediate products is not obvious in the country and the middle and western regions, apart from the eastern region where it shows some technique spillover effects. From the perspective of foreign investment, the overall level and quality of FDI in China by the Belt and Road countries is low and their impact on CO_2_ emissions is not obvious. Taken together, the technique effects of China’s environmental impact on the export trade of the Belt and Road countries mainly depend on the independent technique innovation effects brought by their own R&D investment, and the overall technique effects are negative.

### 6.2. Policy Recommendations

Based on the above analysis, we believe that under the green Belt and Road Initiative, China’s export trade to achieve high-quality green transformation and upgrade can start from the following aspects:

First, further optimize China’s export trade structure to the countries along Belt and Road. On the one hand, we should continue to expand the export market, deeply integrate into the Belt and Road regional value chain cooperation and promote the diversified development of export markets. On the other hand, we need to further increase the investment of R&D and the transformation of scientific and technological achievements, promote the improvement of the ability of independent innovation of enterprises manufacturing technology upgrading, cultivate and develop green and high-tech advantageous industries, increase the technological content and international competitiveness of export products, reduce exports of pollution-intensive products and thereby reduce productive pollution emissions.

In addition, in order to make full use of the capital and talent advantages of the eastern region and play its technological innovation-oriented role. The eastern region, developing industrialization fastest but having the most severe pollution, should speed up to optimize and upgrade export structure for it occupies a dominant position in the Belt and Road export trade. The government and enterprises can establish appropriate incentive mechanisms to encourage and cultivate the development and application of clean technology and high technology to promote the high-quality and green development of export trade in the region. At the same time, this area should form a technology radiation effect to the middle and western regions and even other countries along the Belt and Road and strengthen cooperation with countries in science and technology innovation and jointly build a platform for technological innovation and transfer cooperation. The middle and western regions should pay attention to the experience of development in the eastern region and must not develop the local economy and export trade at the expense of environmental resources. They should cultivate characteristic industries that fit their own advantages to participate in the Belt and Road division of labor and trade cooperation.

Secondly, according to the heterogeneity of economic and trade development and the facts of environmental pollution in various regions in the east, middle, and west, a gradient environmental regulation policy should be implemented. The industrial pollution in the eastern region is the most serious and, given that people’s income levels increase, there are higher requirements for higher environmental quality. Therefore, the government should strengthen the response intensity of environmental regulation policies to rising income levels and then adjust environmental regulation policies to meet development needs. Enterprises should actively respond to the government’s environmental regulations and policies, increase investment in the control of industrial production waste gas pollutants, reduce CO_2_ emissions and achieve sustainable development. The middle and western regions cannot relax environmental regulations. Different levels of environmental regulation policies should be implemented in phases according to the pollution situation in various regions and industries in order to achieve the coordinated development of economic trade and ecological environment.

Furthermore, according to the needs of country’s industrial restructuring and the development of green advantageous industries, we should actively attract foreign investment in advantageous industries and improve the quality and level of foreign direct investment. However, China’s foreign direct investment from countries along the Belt and Road has not shown obvious technology spillovers. With the further development of the Belt and Road Initiative, more countries will participate and industrial cooperation will be broader and deeper, and China should continue to expand the introduction of high-quality, clean foreign capital to obtain technological spillovers, accelerate the green transformation and enterprises upgrading to improve environmental quality and pay attention to avoiding the possibility of undertaking the transfer of highly polluting industries and becoming a “pollution paradise”.

### 6.3. Contributions, Limitations and Future Research Directions

According to the above analysis, existing research mainly focus on empirical studies at the national level. Considering the differences among regions, this paper explores the environmental effects of China’s export trade to the countries along Belt and Road from both national and domestic regional perspectives. Carbon dioxide emissions are used as a representative of pollution emissions to examine the various effects between export trade and pollution emissions. The paper also examines whether the environmental Kuznets curve and pollution haven effect exist. From the research conclusions, it can be seen that the environmental effects of export trade vary significantly among different regions; therefore, this study is a supplement to the existing research. However, due to data limitations, this study did not consider the differences of environmental effects in different industries, especially the impact on carbon emissions, which will make the corresponding environmental protection policy suggestions more targeted; this is a direction for future research. In addition, future studies should also focus on the impact of environmental regulation policies on the environmental effects of export trade and explore the effectiveness and limitations of environmental regulation policies in alleviating negative environmental impacts.

## Figures and Tables

**Table 1:** Heterogeneity regression results of CO_2_ emission effects of China’s export trade to countries along the Belt and Road.

Variables	Ln*C_it_* (Nationwide)	Ln*C_it_* (East)	Ln*C_it_* (Middle)	Ln*C_it_* (West)
LnC*_it_*_−1_	0.335 ***	0.280 ***	0.441 ***	0.374 ***
	(0.183)	(0.195)	(0.237)	(0.116)
Ln*T_it_*	0.976 ***	1.183 ***	0.073 **	0.082 **
	(0.055)	(0.028)	(0.019)	(0.015)
Ln*I_it_*	2.472 **	3.168 ***	2.081 **	1.892 **
	(0.844)	(1.693)	(0.793)	(0.490)
(Ln*I_it_*)^2^	−0.168 **	−0.867 **	0.952	0.513
	(0.078)	(0.094)	(0.145)	(0.036)
Ln*K_it_*/*L_it_*	0.487 **	0.554 ***	0.152 **	0.123 **
	(0.093)	(0.152)	(0.088)	(0.039)
Ln*R_it_*	−2.896 ***	−3.557 ***	−0.414 *	−0.309 *
	(2.201)	(2.593)	(0.737)	(1.056)
*STEI_it_*	−3.035 ***	−5.148 ***	−1.082 ***	−0.827 ***
	(−0.928)	(−1.023)	(−0.886)	(−0.528)
Ln*M_it_*	−0.091	−0.189 *	0.041	0.068
	(0.564)	(1.381)	(0.811)	(0.917)
Ln*FDI_it_*	0.026	−0.019	0.074	0.037
	(1.062)	(0.966)	(0.410)	(0.547)
*β*_0_	−16.571 ***	−15.338 ***	4.364 *	−6.425 **
	(7.596)	(2.919)	(0.452)	(1.532)
AR (1)	−0.028 ***	−0.045 ***	−0.092 ***	−0.087 ***
AR (2)	0.184	0.272	0.199	0.136
Hausman test	1.623	1.307	1.482	1.216
Obs	210	70	63	77

Notes: Values in parentheses are *t* values; ***, **, * indicate significant levels at 1%, 5% and 10%, respectively.

## Data Availability

Not applicable.
